# UV radiation promotes anthocyanins biosynthesis in the fruit peel of blood oranges (*Citrus sinensis*)

**DOI:** 10.3389/fpls.2025.1679102

**Published:** 2025-09-25

**Authors:** Haijian Yang, Hao Chen, Wu Wang, Shuang Li, Min Wang, Lin Hong, Lei Yang, Wei Hu

**Affiliations:** Chongqing Academy of Agricultural Sciences, Chongqing, China

**Keywords:** blood orange, UV light, anthocyanin, transcriptome, metabolome, glycosyltransferase

## Abstract

**Introduction:**

The commercial value of blood oranges (*Citrus sinensis*) is closely linked to the intensity of red pigmentation in the peel and flesh, driven by the accumulation of anthocyanins. While light is a crucial environmental factor for anthocyanin synthesis, the specific effects of different light spectra, particularly ultraviolet (UV) radiation, on peel pigmentation have not been fully elucidated.

**Methods:**

In this study, the effects of light spectra on anthocyanin biosynthesis in blood orange peel were systematically studied through three treatments of visible light (VL), UV and complete shading (CK). These treatments were combined with transcriptome, anthocyanin targeted metabolome and weighted gene coexpression network analysis (WGCNA).

**Results and Discussion:**

After 40 days, UV-treated fruit exhibited significantly higher anthocyanin content and color index (CI) than other treatments, with a significantly positive correlation between the two. Metabolomics identified four key anthocyanins, including cyanidin-3-o-glucoside and its 2 derivatives, as the primary contributors to pericarp coloration, with their levels significantly increased under UV exposure. WGCNA screened three core gene modules closely associated with anthocyanin metabolism, and further identified three glycosyltransferase genes (ugt79b1, bz1 and GT1) as hub genes involved in anthocyanin accumulation. This study demonstrates that UV light enhanced anthocyanin synthesis in blood orange peel by activating downstream glycosylation pathways, providing both a theoretical basis and technical approach for improving commercially market value of blood orange through light regulation.

## 1 Introduction

Blood oranges are rich in anthocyanins, which give the fruit its red color and offer strong antioxidant properties that benefit human health (Ma et al., 2021; Sadowska-Bartosz and Bartosz, 2024; Yang et al., 2023b), making them a popular choice. In Southwest China, blood orange fruits usually reach maturity between February and March in production. However, leaving them on the tree over winter increases the risk of freezing damage. Hence, the fruits are typically harvested in December, which limits anthocyanin accumulation and leads to poor coloration. This significantly affects their commercial value and market competitiveness (Liu et al., 2024). Therefore, seeking efficient and safe ways to promote anthocyanin accumulation in blood orange peels is of great importance for enhancing their commercial value.

The biosynthesis of anthocyanins is a complex and precisely regulated metabolic process. This pathway begins with phenylalanine, and proceeds through a series of enzymatic reactions to generate anthocyanin aglycogenes, which are further modified to form stable anthocyanins through glycosylation (An et al., 2019; Zhang et al., 2014). This pathway involves a series of structural genes encoding key enzymes, including phenylalanine aminolase (PAL), Chalketone synthase (CHS), chalketone isomerase (CHI), flavanone-3-hydroxylase (F3H), dihydroflavonol-4-reductase (DFR), and anthocyanin synthase (ANS), etc (Holton and Cornish, 1995). Unlike many genes in secondary metabolic pathways that are organized into Biosynthetic Gene Clusters (BGCs), the structural genes of anthocyanin biosynthesis are usually scattered throughout different positions in the genome. This indicates that their regulation is more complex and relies on multiple coordinated regulatory factors (Qiao et al., 2025). At the transcriptional level, expression of these structural genes is strictly regulated by a transcriptional complex, composed of R2R3-MYB, bHLH and WD40 repeat proteins, which is usually referred to as the MBW complex (Walker et al., 1999). Among these, MYB transcription factors are usually the key components that determine regulatory specificity. They can recognize and bind to specific cis-acting elements in the promoter regions of downstream structural genes, thereby activating their transcription (Takos et al., 2006; Yan et al., 2021). bHLH and WD40 proteins, acting as cofactors, can enhance the stability and activity of the transcriptional activation complex through interacting with MYB protein (Mathews et al., 2003). In addition to their core positive regulatory module, plants have also evolved negative regulators to achieve precise spatiotemporal control of anthocyanin synthesis (LaFountain and Yuan, 2021). These negative regulators may form a complex “double negative” regulatory logic by competing with MBW complex or directly inhibit the transcription of structural genes, ensuring that anthocyanins accumulate in appropriate amounts only at specific developmental stages and under specific environmental conditions (LaFountain and Yuan, 2021).

The biosynthesis of anthocyanins is regulated by various endogenous and exogenous signals, including developmental signals, plant hormones and environmental factors. Among various environmental factors, light is one of the most crucial factors regulating anthocyanin synthesis (Guo et al., 2008). Within the light spectrum, ultraviolet (UV) radiation, particularly UV-B (280–315 nm) and UV-A (315–400 nm), is a potent signal for inducing anthocyanin accumulation (Li et al., 2021). UV-B is mainly perceived by the UVR8 (UV RESISTANCE LOCUS 8) photoreceptor, while UV-A is mainly perceived by Cryptochromes (CRYs). These optical signals are transmitted through downstream signal elements such as Constitutive Photomorphogenic 1 (COP1) and Elongated Hypocotyl 5 (HY5) (Zhang et al., 2022). HY5 is a bZIP transcription factor that plays a core role in optical signal transduction. It can directly bind to the promoters of structural and regulatory genes (such as CHS, DFR and MYB) involved in anthocyanin synthesis, promoting their expression (Zhao et al., 2021). A large number of studies have shown that UV radiation is a key factor in promoting the coloring of fruit peels (An et al., 2019; Chen et al., 2024; Henry-Kirk et al., 2018; Li et al., 2020; Zhou et al., 2007). However, most existing studies focus on the overall effects of light vs. no light (such as bagging treatment) and rarely distinguish the independent roles of visible light (VL) and ultraviolet (UV) in regulating anthocyanin synthesis in blood orange peels.

To address this gap, this study designed three light treatments, including complete shading (CK), visible light source (VL), and ultraviolet light (UV). Using targeted metabolomics and transcriptomics analytical techniques, this study aims to systematically analyze the specific regulatory effect of UV radiation on anthocyanin biosynthesis in the peel of blood orange fruits during color development. The findings will improve our understanding of how environmental factors regulate citrus fruit quality and provide important theoretical basis and a technical reference for improving the nutritional value and commercial traits of blood oranges through optimizing light management.

## 2 Materials and methods

### 2.1 Experimental materials and design

Seven-year-old ‘Tarocco’ blood orange (*Citrus sinensis* L. Osbeck ‘Tarocco’) trees from the Zhenwu citrus base, Fruit Tree Institute, Chongqing Academy of Agricultural Sciences, were used in the study. Thirty uniform trees were selected, with ten as the CK, ten treated with VL exposure and ten with UV. During the peel coloration period, shading nets are used to block external light. Subsequently, the fruit surfaces were exposed to either VL (white light 400–700 nm) (Sun et al., 2024) or UV light (UV-A, 315–400 nm and UV-B, 280–315 nm) and the light intensity was set at 540 μmol/m²/s (**Supplementary Figure S1**). The plants subjected to VL and UV treatments were maintained under a photoperiod consisting of 16 hours of light followed by 8 hours of darkness. The unexposed fruits served as the CK. Samples were taken every 20 days after the start of treatment. For each treatment, five fruits were randomly selected from the tree canopy periphery, with three replicates. After sampling, the outer layers of the fruit peels were peeled off and frozen in liquid nitrogen for storage at -80°C.

### 2.2 Determination of the appearance color of fruit peel

The CI value of the peel was determined as described in our previous study (Hu et al., 2021). A Chroma Meter CR-400 Spectrophotometer was used to obtain CIE L^*^, a^*^, and b^*^ values at three different points on the peel. The CI was calculated using the formula:


CI=1000×a/(L×b)

### 2.3 Determination of the anthocyanin content in fruit peel

Anthocyanin content in fruit peels was determined by spectrophotometry (Jin et al., 2023), and calculated using the formula:


$$\begin{array}{c} \text{Anthocyanin content }\\ \left({\text{g kg}}^{-1}\text{FW}\right)=\left(\text{A}510\text{ at pH}1.0\;–\text{ A}510\text{ at pH}4.5\right)\\ \times 484.8\left(\text{molecular weight of cyanidin}\right)/24,825\\ \left(\text{molar absorption coefficient of cyanidin at A}510\right)\times \text{dilution ratio} \end{array}$$

### 2.4 Anthocyanin-targeted metabolome analysis

This analysis was performed at MetWare Biotechnology in Wuhan, China, according to standard protocols (Yuan et al., 2018). Data reliability was assessed through quality control (QC). Statistical analysis of the data matrices containing the metabolite measurement values was performed using Analyst software (version 1.6.1). The hierarchical cluster analysis was used to generate heatmaps with dendrograms for the samples and metabolites.

### 2.5 Illumina transcriptomic sequencing

Total RNA of each sample was extracted using TRIzol reagent (Invitrogen, Thermo Fisher, MA, USA) following the manufacturer’s protocol. Both integrity number of RNA (RIN) and its contamination were determined by the Agilent 2100 Bioanalyzer system (Agilent Technologies, CA, USA) and 1% agarose gels. Sequencing libraries were generated as follows. The mRNA was purified from total RNA using poly-T oligo-attached magnetic beads. Fragmentation was carried out using divalent cations under elevated temperature in an Illumina proprietary fragmentation buffer. First strand cDNA was synthesized using random oligonucleotides and Super Script II. Second strand cDNA synthesis was subsequently performed using DNA Polymerase I and RNase H. Remaining overhangs were converted into blunt ends via exonuclease/polymerase activities and then the enzymes were removed. Libraries were sequenced on a six-lanes HiSeq 2500 System (Illumina) according to the SR60 protocol. Poly-N, reads with adapters, and low-quality reads were eliminated from the obtained raw data. The fragments per kilobase of exon model per million mapped fragments (FPKM) and gene alignment of each gene regarding its length were determined by FeatureCounts v1.6.2 (Liao et al., 2014). Differential gene expression between the two classes of the samples were analyzed using DESeq2 v1.22.1. P values was adjusted using the Benjamini & Hochberg method. The thresholds of considerable differential expression were indicated by |log2foldchange| and corrected P values. The false discovery rate (FDR) approach was employed to evaluate the importance of gene expression differences using threshold P value of various tests. Genes with FDR<0.05 and |log2Fold Change|≥1 were considered significantly differentially expresses.

### 2.6 Application of WGCNA in metabolite and transcriptome analysis

The R package WGCNA was used to construct the co-expression network modules from gene expression data and then correlate these modules with metabolite abundances. The modules were based on the topological overlap measure (TOM) using the default parameters of the automatic network construction function (blockwiseModules). Initial clusters were merged based on eigengenes, and the eigengene value was calculated for each module to identify its associations with anthocyanin-related metabolites.

### 2.7 RNA−seq data validation by qRT-PCR

A SYBR Green system (TaKaRa, Dalian, China) was used to conduct the quantitative real-time polymerase chain reaction (qRT-PCR). The samples were cycled 35 times by being heated at 95°C for 5 min for predenaturation, followed by at 94°C for 30 sec, 56°C for 30 sec, and 72°C for 90 sec. The primers were designed using Primer Premier 5.0 software (Premier Biosoft, CA, USA). A full list of all the primers is presented in **Supplementary Table S5**. Relative gene expression levels were determined according to the 2^−ΔΔCt^ approach. All procedures included three independent technical and biological replicates.

## 3 Results

### 3.1 Effects of UV light on fruit peel color and anthocyanin accumulation in blood oranges

**Figure 1:**
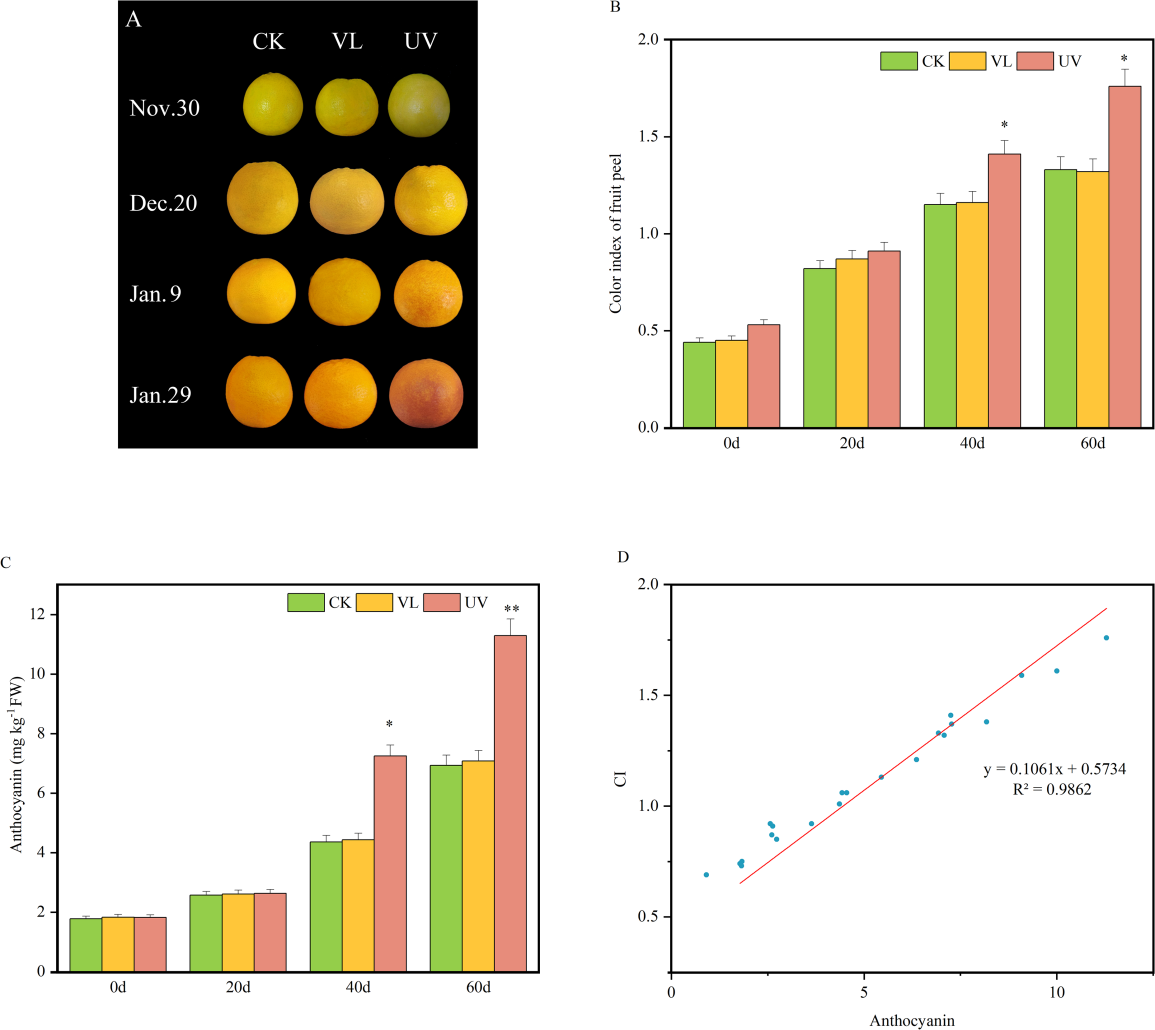
Dynamic changes in peel color and anthocyanin content of blood oranges for different light treatment. **(A)** Phenotypic characteristics of fruit peel at various developmental stages. **(B)** Color index of fruit peel. **(C)** The content of anthocyanins in fruit peels. **(D)** Correlation analysis between fruit peel color index (CI) and anthocyanin content. * and ** indicate significant differences.* represent P<0.05, ** represent P<0.01.

After 20 days of light treatments, there were no significant differences in fruit phenotypes and color index (CI). However, after 40 days, fruit peels under UV treatment showed significant color change compared to the other treatments. Meanwhile, the CI of the UV treatment was also significantly higher than that of the VL and CK treatments (**Figures 1A, B**). Correspondingly, anthocyanin content was significantly higher in UV-treated fruit peels than in VL and CK after 40 days. Regression analysis a significant positive correlation between peel CI and anthocyanin content, indicating that UV treatment promotes both coloration and anthocyanins accumulation in blood orange peels.

### 3.2 Sequencing data statistics

RNA sequencing produced 1,692,161,564 clean reads, with an average of 47,004,487 reads per sample (**Supplementary Figure S2A**). Correlation analysis showed a strong correlation among biological duplicate samples at each treatment times (**Supplementary Figure S2B**). An average Q30 value of 95.16% was recorded across the 36samples, ranging from 94.53% to 95.66%, confirming high quality of the sequencing data (**Supplementary Table S1**).

### 3.3 Transcriptomic analysis of fruit peel under different treatment

**Figure 2:**
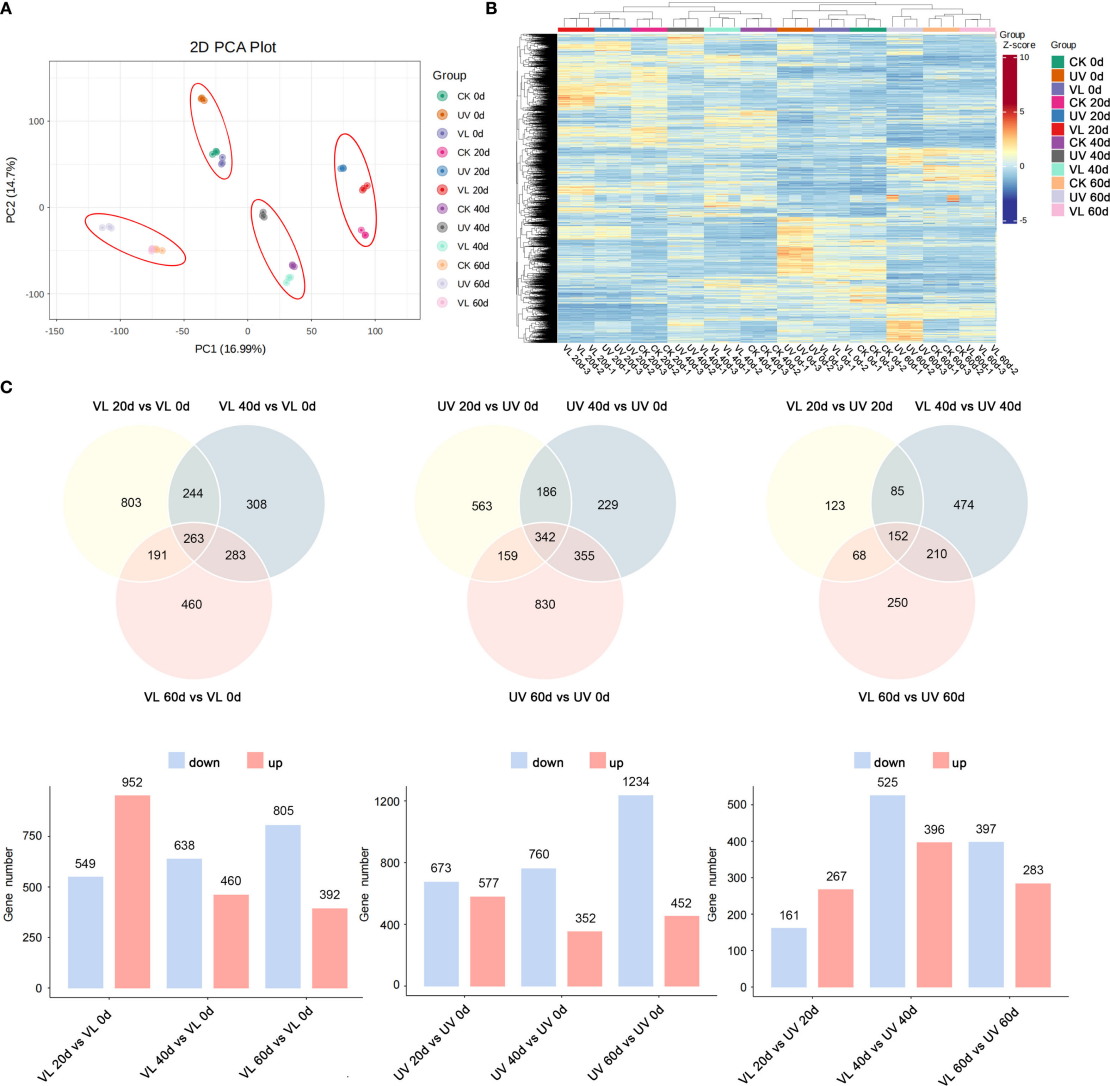
Transcriptomic profiles of fruit peels in response to various light treatments at four phase of blood orange growth stage. **(A)** Principal component analysis (PCA) based on FPKM data. **(B)** The expression of DEGs among different samples. **(C)** Comparative analysis of DEGs in treatment of VL and UV at different periods.

In the PCA analysis, PC1 and PC2 accounted for 16.99% and 14.7%, respectively, of total variation in gene expression among all samples, indicating the transcriptome data’s strong discriminatory power (**Figure 2A**). The heat map generated from hierarchical cluster analysis revealed distinct temporal gene expression patterns, with UV-treated samples displaying opposite trends compared to CK and VL treatments at some point in time (**Figure 2B**). A total of 5,470 differentially expressed genes (DEGs) were identified across the three treatments (CK, VL, UV) and four sampling time points (**Supplementary Table S2**). In the VL treatment, a total of 1,501, 1,901 and 1,197 DEGs were detected at 20 d, 40 d and 60 d, respectively. In the UV treatment, 1,250, 1,112, and 1,686 DEGs were found after 20 d, 40 d, and 60 d, respectively (**Supplementary Table S3**). In this study, a total of 1,362 DEGs were identified in response to the VL and UV treatments. The number of DEGs ranged from 68 to 474 across different treatment times, with 152 DEGs being coexpressed (**Figure 2C**).

### 3.4 Clustering and functional analysis of separate developmental periods

**Figure 3:**
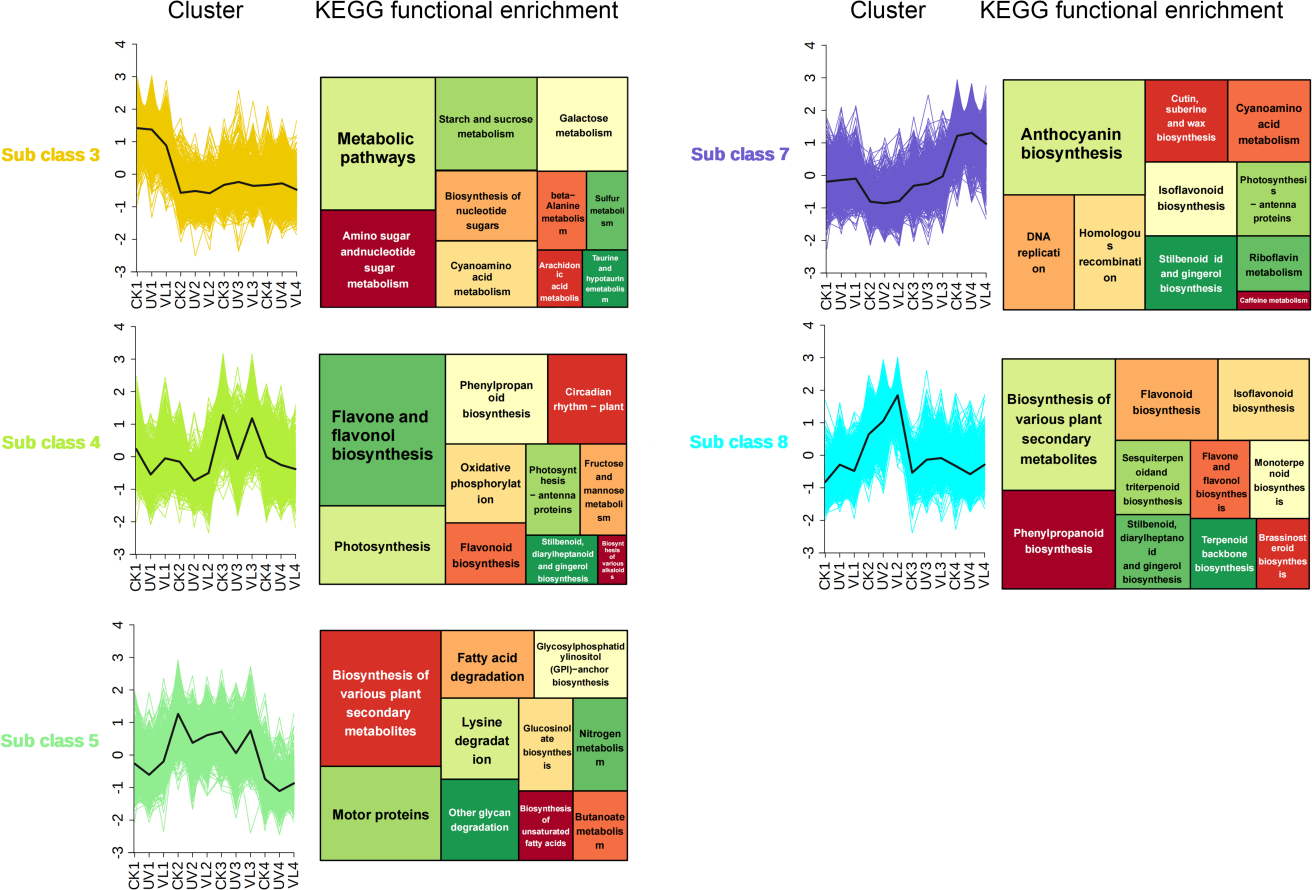
K-means cluster and KEGG functional analysis of DEGs at four phase of blood orange growth stage for different light treatment. Within the first column, the DEG k-means cluster analysis showcases modules exhibiting significantly divergent trends. The second column highlights the results of the KEGG enrichment analysis conducted on genes within these modules. CK1-CK4 means CK 0d-CK 60d. Treatments in VL and UV are also similar.

The dynamic variations in expression profiles across light treatments exhibited characteristics of time series data. Using k-means clustering on all DEGs, 8 subgroups of DEGs were identified, with 5 subgroups (subgroup 3, 4, 5, 7, 8) exhibiting contrasting trends at various time points (**Figure 3**). KEGG functional enrichment analysis in the subgroups revealed that DEGs in subgroups 3, 4 and 7 were mainly involved in metabolic pathways, flavonoid and flavonol biosynthesis, and anthocyanin synthesis, respectively. DEGs in subgroups 5 and 8 were enriched in pathways related to plant secondary metabolites. It is worth noting that subgroup 7, which is mainly enriched in anthocyanin synthesis, was down-regulated in the 0–20 days after UV treatment, but up-regulated in the 40–60 days, aligning with the observed anthocyanin accumulation trend.

### 3.5 Targeted metabolism analysis of anthocyanins in fruit peel

**Figure 4:**
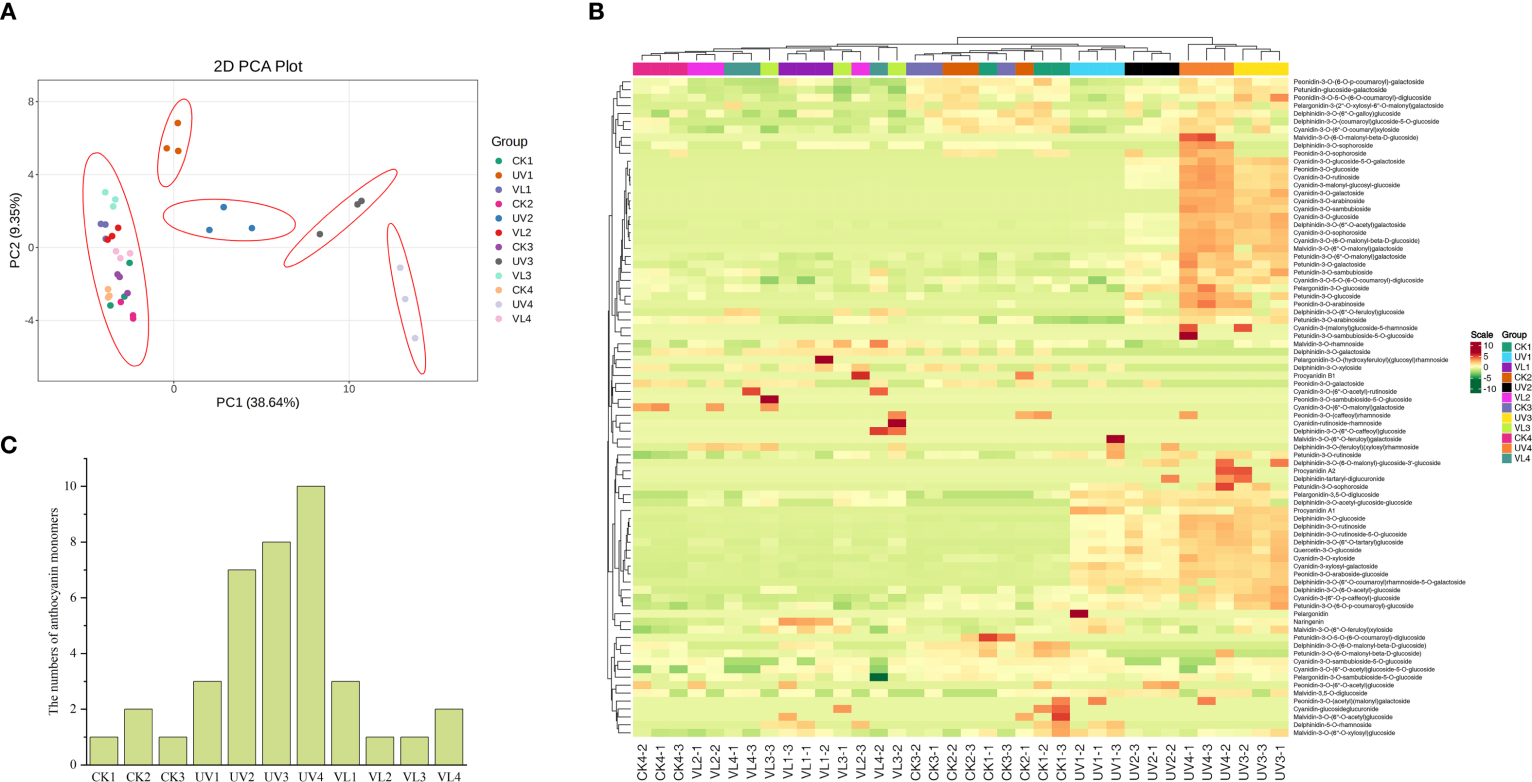
Anthocyanin metabolic profiles of fruit peels during four blood orange developmental stages. **(A)** PCA of anthocyanin contents identified in all samples. **(B)** Cluster heatmap of anthocyanin targeted metabolites and all samples. The cluster lines on the left are metabolite cluster lines, and those above are sample cluster lines. **(C)** The number of anthocyanin monomers in the sample (anthocyanin-related metabolites with a content > 1μg g^-1^). CK1-CK4 means CK 0d-CK 60d. Treatments in VL and UV are also similar.

Through a targeted database containing 83 anthocyanin metabolism-related compounds, we obtained the anthocyanin metabolome data of blood orange peels with different treatment (**Supplementary Table S4**). The PCA analysis showed that the UV-treated samples at each sampling time points were well separated, while the VL and CK samples clustered together, likely due to their low metabolite levels (**Figure 4A**). Cluster analysis of all samples and targeted metabolites confirmed that UV treatment after 40 days of light exposure formed a distinct group, whereas the other two treatments fall into a separate category (**Figure 4B**). This classification aligned with results of anthocyanin content (**Figure 1C**). In order to identify the anthocyanin compounds that were most closely related to the coloring of blood orange peel, anthocyanin-related metabolites with levels above 1μg g^-1^ in all samples were screened out. The results showed that a total of 10 anthocyanin compounds were identified in only UV treated samples at 60 d (**Figure 4C**). Meanwhile, the anthocyanin compounds identified in UV treatment are also much higher than those in other treatments. Among all the samples, Quercetin-3-O-glucoside had the highest content, followed by Cyanidin-3-O-(6-O-malonyl-β-D-glucoside), cyanidin-3-O-sophoroside, Cyanidin-3-malonyl-glucosyl-glucoside and cyanidin-3-O-glucoside (**Supplementary Figure S3**). While Quercetin and its glycosides were widely present and considered as important antioxidants and secondary metabolites, they did not directly contribute to coloration in blood oranges. Instead, cyanidin glycoside derivative was the dominant coloring compound.

### 3.6 Profiling the expression of transcription factors implicated in anthocyanin biosynthesis pathways

**Figure 5:**
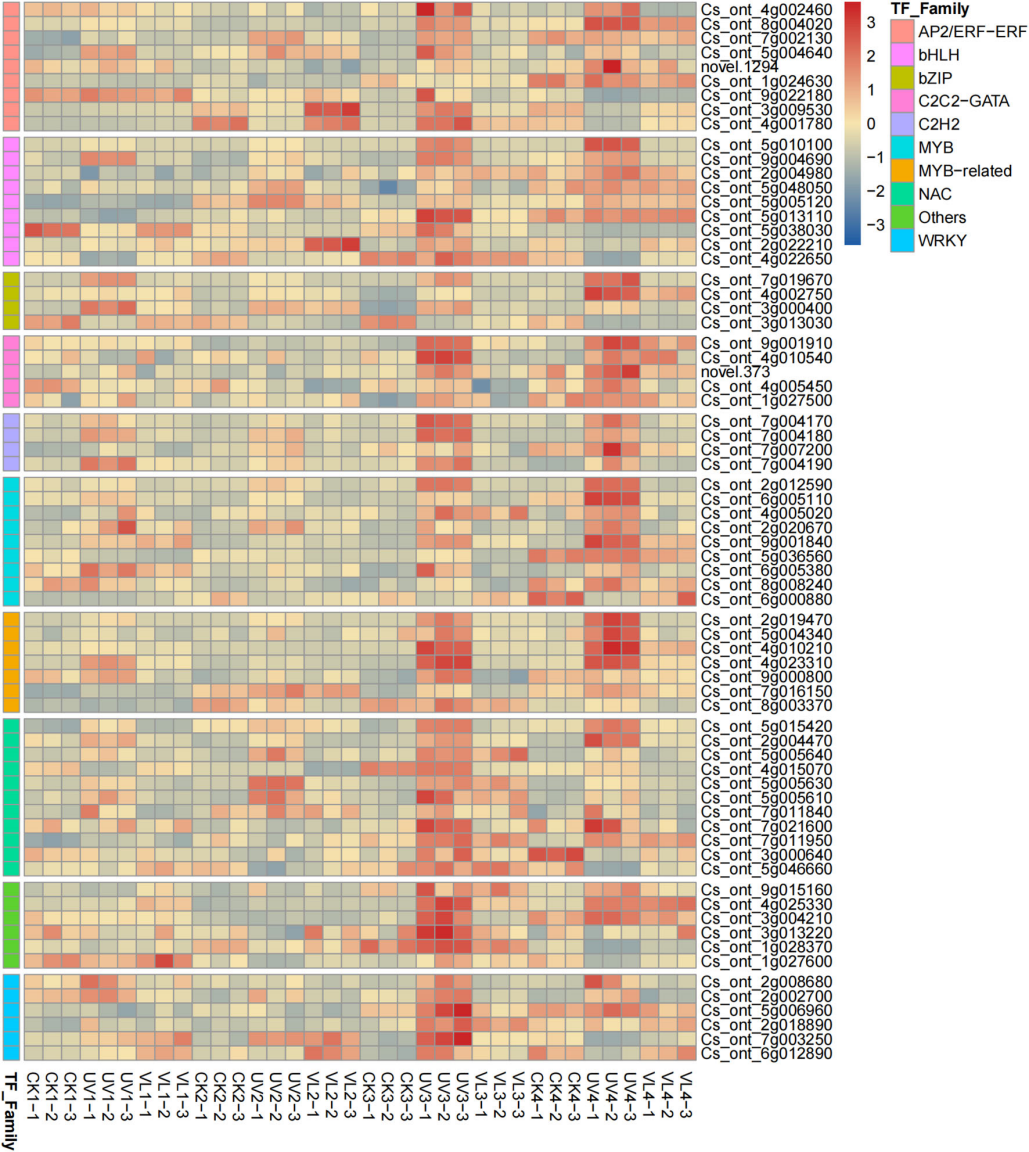
Heatmap diagrams of TFs involved in anthocyanin pathways(Top 10). The color scale bar at the right side denotes the average FPKM value that has been log-transformed. CK1-CK4 means CK 0d-CK 60d. Treatments in VL and UV are also similar.

A total of 1,513 TFs with different expression abundances were detected, with 122 related to anthocyanin metabolism being classified into 40 families. Among these, NAC (11), AP2/ERF-ERF (9), MYB (9), and bHLH (9) were most prevalent. The UV treated samples at 40 d and 60 d had significantly higher TF expression levels other treatments (**Figure 5**).

### 3.7 Co-expression network analysis of weighted genes

**Figure 6:**
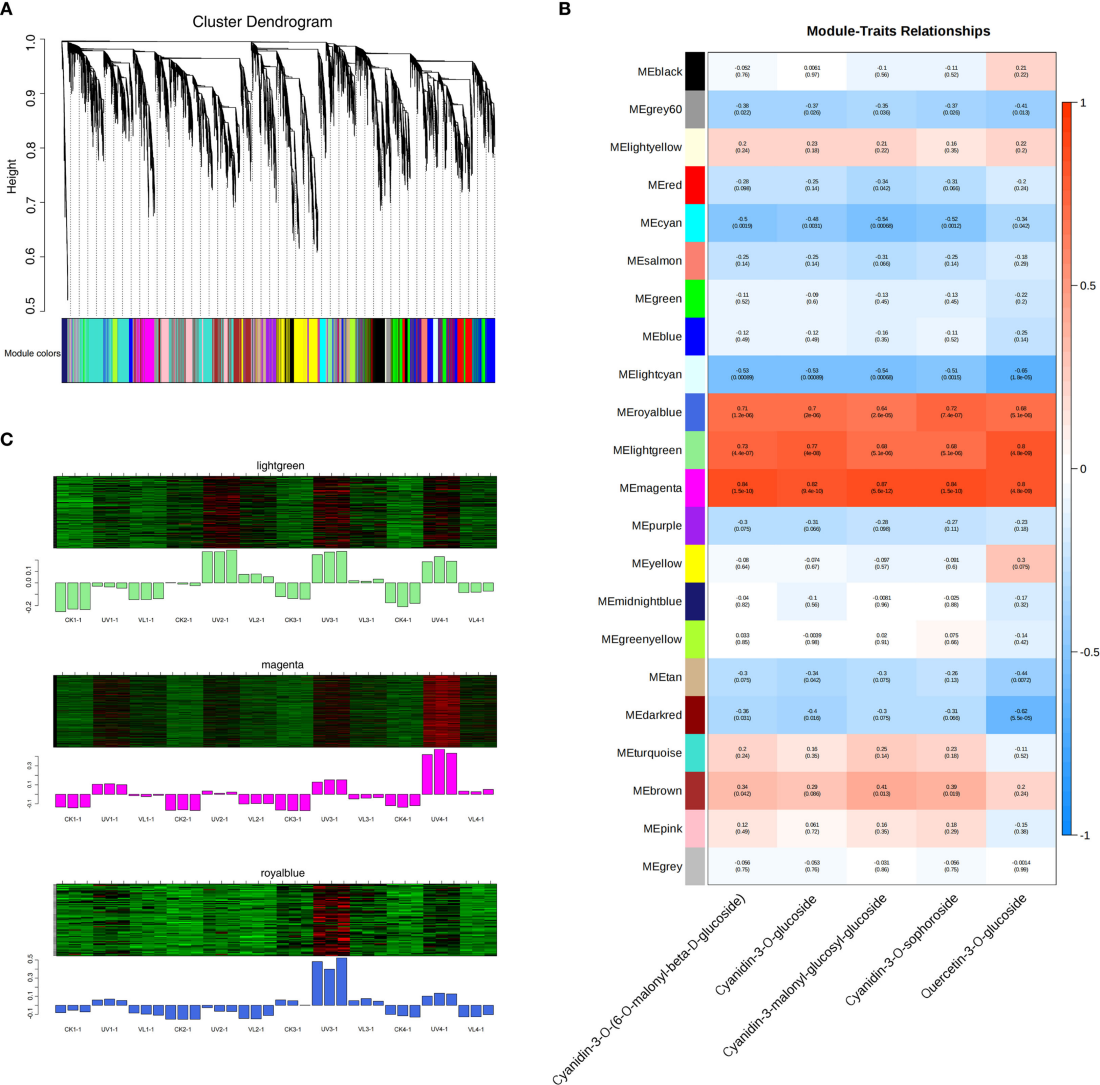
Gene networks involved in fruit peel anthocyanin metabolism during blood orange development as identified by WGCNA. **(A)** A cluster dendrogram illustrates 21 modules of co-expressed genes identified through WGCNA. The main tree branches represent the 21 modules, distinguished by various colors. **(B)**The relationship between modules and the major anthocyanin metabolism compounds is illustrated, with each row representing a module and each column representing a chemical compound. Heatmap colors represent the correlation level, the numbers out and in parentheses are correlation coefficient r and P value, respectively. **(C)** Pattern diagram of module gene expression. Upper, the heat map of gene clustering within the module. Lower, the expression patterns of module feature values across different samples. CK1-CK4 means CK 0d-CK 60d. Treatments in VL and UV are also similar.

The weighted gene co-expression network analysis (WGCNA) was performed to analyze the co-expression networks of the identified DEGs involved in anthocyanin metabolism. Subsequent to the filtration of deletion and outlier values, a total of 17,562 genes were preserved across all treatments. The genes were categorized into 21 color-coded modules, each denoted by a specific color, indicating significant correlations among the genes within each module. According to the analysis results of targeted metabolomics, the primary anthocyanin metabolites were identified. Then, ANOVA was carried out for each anthocyanin metabolite to examine the significant variations among the treatments (p< 0.05). If there existed a significant difference among the treatments, these anthocyanin metabiolates were selected for subsequently WGCNA analysis (**Figure 6A**). Pearson correlation analysis identified three modules (lightgreen, magenta and royalblue) significantly associated with the anthocyanin metabolism compounds (Cyanidin-3-O-(6-O-malonyl-β-D-glucoside), Cyanidin-3-O-glucoside, Cyanidin-3-malonyl-glucosyl-glucoside, Cyanidin-3-O-sophoroside and Quercetin-3-O-glucoside) (**Figure 6B**). The UV treatment upregulated most genes in all modules (**Figure 6C**). KEGG enrichment analysis on the genes involved in these three modules revealed that the characteristic genes located in the top 20 pathways were mainly involved in amino acid metabolism, secondary metabolite synthesis, and carbohydrate metabolism pathways (**Supplementary Figure S4**). In addition, these three modules were also enriched with genes related to photosynthesis, plant hormone signal transduction, flavonoid biosynthesis and circadian rhythm-plant.

**Figure 7:**
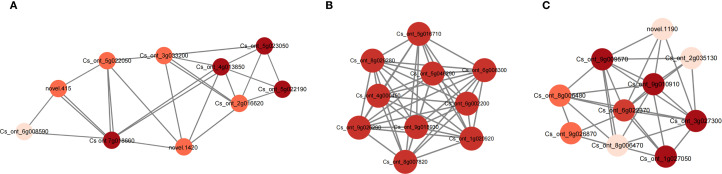
The network diagrams of key candidate genes involved in anthocyanin metabolism process in **(A)** lightgreen, **(B)** magenta and **(C)** royalblue modules. The depth of the dot color indicates the degree of connectivity of this gene with other genes within the network.

To identify key regulators, the top 10 hub genes were selected to establish the relative correlation networks within the lightgreen, magenta, and royalblue modules (**Figure 7**). Among these modules, three genes were identified, namely anthocyanidin 3-O-glucoside 2’’’-O-xylosyltransferase (UGT79B1, Cs_ont_4g013850) in the lightgreen module, anthocyanidin 3-O-glucosyltransferase (BZ1, Cs_ont_5g016710) in the magenta module, and anthocyanidin 5,3-O-glucosyltransferase (GT1, Cs_ont_9g026870) in the royalblue module, suggesting all these genes playing important roles in the anthocyanin metabolism process during the coloring of blood orange peels.

### 3.8 qRT-PCR validation

The RNA-seq results were validated through examining ten randomly selected DEGs, as indicated by the transcriptome analysis. The relative expression levels (2^−ΔΔCt^) of the DEGs determined by qPCR aligned well with the transcriptome data (FPKM), as illustrated in **Supplementary Figure S5**. There was a significantly positive correlation (R^2^ = 0.9317) between these two, confirming the exceptional reliability of the RNA-seq data.

## 4 Discussion

Peel color is a key factor determining the commercial value of blood oranges and consumer preferences. The unique red color of blood oranges mainly results from the accumulation of anthocyanins. This study found that compared with VL and CK, UV light exposure significantly promoted peel coloration and anthocyanin accumulation, with a strong positive correlation between the peel color index (CI) and anthocyanin content (Ban et al., 2007). These results are consistent with previous findings in different plant species, such as blueberries (Li et al., 2021), grapes (Berli et al., 2011), mangoes (Yang et al., 2024) and lettuce (Skowron et al., 2024), where UV radiation effectively promoted anthocyanins synthesis. Anthocyanin accumulation in plants in response to UV stress is an important protective mechanism. Anthocyanins and other flavonoids can absorb high-energy UV band (280–315 nm) radiation, protecting sensitive intracellular components such as chloroplasts and DNA from damage (Agati and Tattini, 2010). In addition, UV stress induces the production of reactive oxygen species (ROS), and anthocyanins as effective antioxidants, which can eliminate excessive ROS and reduce oxidative damage (Yang et al., 2024). In this study, anthocyanin content significantly increased after 40 days of UV treatment, but not at 20 days, suggesting that UV-induced anthocyanin accumulation is a progressive and cumulative process requiring continuous stimulation (Li et al., 2020).

The main chromogenic anthocyanins identified in this study, cyanidin-3-O-glucoside, cyanidin-3-O-sophoroside, and cyanidin-3-O - (6-O-malonyl - β - D-glucoside), are consistent with previous findings in various red or purple plant tissues (Memete et al., 2022). For example, in sweet cherries, mulberries, blackthorn plums, and some colored grains, cyanidin-3-O-glucoside or its rutin glycoside has been shown to be the main coloring anthocyanin (Crupi et al., 2014; Huang et al., 2020; Kim et al., 2007; Velickovic et al., 2014). Therefore, our study links the coloring mechanism of blood oranges to the broader biochemical background of plant pigments, confirming that blood oranges follow a conserved coloration strategy within the plant kingdom. Further, the presence of malonylated anthocyanin detected in this study, namely cyanidin-3-O-(6-O-malonyl-β-D-glucoside), suggest that blood orange peel not only synthesizes basic anthocyanins but also refines its structure through subsequent enzymatic reactions to achieve a more enduring and intense color presentation.

A significant finding is that the highest content of “anthocyanin related metabolites” is actually quercetin-3-O-glucoside, a flavonol rather than anthocyanin. Flavonoids are usually colorless or pale yellow, and do not directly contribute to red coloration. However, their high abundance suggests a key non-chromogenic role in the physiology of fruit peels. First, flavonols are highly efficient UV-B absorbers and play core roles in light protection of plants (Agati and Tattini, 2010). UV-B radiation exposure can induce plants to produce large amounts of reactive oxygen species (ROS) (Shoaib et al., 2024), and flavonols can not only reduce the penetration of ROS into sensitive targets inside cells by absorbing UV-B radiation (Agati et al., 2021), but also directly eliminate ROS to alleviate oxidative damage (Nakabayashi et al., 2014). Second, flavonols function as copigments. They can form complexes with anthocyanin molecules through hydrophobic interaction and hydrogen bonds, which enhance the color intensity of anthocyanins (color-enhancing effect) and shift their color from red to purple or blue (redshift effect), while improving stability of anthocyanins in aqueous solutions (Wang et al., 2024). Therefore, the high level of quercetin-3-O-glucoside in the skin of blood oranges likely interacts with anthocyanins to shape the unique, stable, and saturated red coloration in blood orange peel.

In this study, we simplified the complex DEGs dataset into 21 functional modules through WGCNA and successfully identified the three modules that were most relevant to changes in anthocyanin metabolite content. This not only significantly narrowed the pool of candidate genes but also revealed the intrinsic functional connections among these genes, consistent the recent findings in other citrus fruits (Jin et al., 2023). The KEGG enrichment of the key modules revealed strong connections between anthocyanin synthesis and broader metabolic pathways, including amino acid metabolism, secondary metabolite synthesis and carbohydrate metabolism. Phenylalanine serves as the starting substrate of the phenylpropanoid pathway, and directly influences the biosynthetic flux of the entire flavonoid pathway, including anthocyanins (Shao et al., 2025). Carbohydrate metabolism not only provides energy (ATP) and reducing power (NADPH) for the synthesis process, but more importantly, provides a sugar donor (such as UDP glucose) which is necessary for anthocyanin glycosylation modification (Lin et al., 2025). These results indicate that the efficient anthocyanins accumulation in blood orange peel is coordinated by both primary and secondary metabolisms. Through such precise and integrated network regulation, cells ensure adequate supplies of synthetic precursors, energy and modification groups. The integration and regulation of this metabolic flow are common in plant pigment formation (Jia et al., 2025) and stress response (Song et al., 2025).

In addition to the identification of key glycosyltransferase genes, our results also highlight the regulatory role of transcription factors (TFs) in shaping anthocyanin biosynthesis and peel coloration. In particular, the expression of MYB, bHLH, and WD40 family members was significantly up-regulated under UV treatment, which is consistent with their roles in forming the MBW transcriptional complex that directly activates structural genes such as CHS, DFR, and ANS. The enhanced activity of this regulatory complex leads to increased production of cyanidin-based anthocyanins, including cyanidin-3-O-glucoside and its malonylated derivatives, which are the major pigments contributing to the red coloration of blood orange peel. Therefore, the regulatory framework in our study can be conceptualized as a “Transcription factors–Genes–Metabolites–Colors” axis (**Supplementary Figure S6**). Along this axis, TFs (e.g., MYB, bHLH, and WD40) regulate the expression of structural genes, which determine the synthesis and accumulation of anthocyanin metabolites. The qualitative and quantitative composition of these metabolites then directly translates into the observed peel colors.

A key outcome of this study was the identification of three glycosyltransferase (UGTs) genes as hub genes in the key module. Along the anthocyanin synthesis pathway, glycosylation is the final crucial modification step, and thus it determines stability, solubility, color tone and biological activity of anthocyanins by attaching sugar groups (such as glucose, galactose, xylose, etc.) to anthocyanins (Zhang et al., 2023). The BZ1 (anthocyanin 3-O-glucosyltransferase) and GT1 (anthocyanin 5,3-O-glucosyltransferase) identified in this study catalyze the glycosylation at the C3 and C5 positions, forming stable anthocyanin structure (Fierri et al., 2023). The identification of UGT79B1 (3-O-glucoside 2’’’-O-xylosyltransferase) further revealed complex glycosylation modifications in blood oranges, adding xylose to glucose to form more diverse polysaccharide chains. This modification is key to the formation of species-specific anthocyanin profiles in plants (Wang et al., 2023). Previous studies on blood orange pulp have mostly focused on upstream structural genes of anthocyanin synthesis pathways (such as CHS, DFR, ANS), and their regulating transcription factors (such as MYB, bHLH, WD40 complex) (Yang et al., 2023a, 2023c). This study identified these UGTs genes as “hubs” in the network through WGCNA, demonstrating that in blood orange peel, the downstream glycosylation modification step is the core regulatory node for the final accumulation and variety diversity of anthocyanins. This provides a new perspective on the fine regulation of anthocyanin synthesis, being that once the synthetic pathway is initiated by transcription factors, the differential expression of UGTs genes determines the end product’s structure and diversity.

## 5 Conclusions

This study demonstrates that UV light significantly promotes anthocyanin accumulation and enhances peel coloring of blood orange. Key anthocyanin metabolites including cyanidin-3-O-glucoside, cyanidin-3-O-sophoroside, cyanidin-3-malonyl-glucosyl-glucoside, and cyanidin-3-O-(6-O-malonyl-β-D-glucoside) were identified as primary ones directly related to anthocyanin biosynthesis and peel coloration. Transcriptomic analysis revealed significant differences in gene expression patterns under UV treatment, particularly in genes involved in flavonoid biosynthesis pathways. Network analysis identified three glycosyltransferase (UGT) genes—UGT79B1, BZ1, and GT1—as critical regulators of anthocyanin diversity and accumulation via complex glycosylation modifications. Importantly, unlike fruit pulp, anthocyanin biosynthesis in the fruit peel involves an additional dimension of finely tuned regulation through glycosylation modifications. These findings offer valuable insights for developing cultivation strategies that utilize targeted UV treatments to improve the market value of blood oranges.

## Data Availability

The data presented in the study are deposited in the Mendeley Data repository. (https://doi.org/10.17632/8dwmcy55x6.1).
