# Supplementary material for: UV radiation promotes anthocyanins biosynthesis in the fruit peel of blood oranges (Citrus sinensis)
Source: Front Plant Sci. 2025 Sep 25;16:1679102. doi: 10.3389/fpls.2025.1679102 (PMC12507625; doi:10.3389/fpls.2025.1679102)
Supplement: Supplementary file 1 [file DataSheet1.doc]

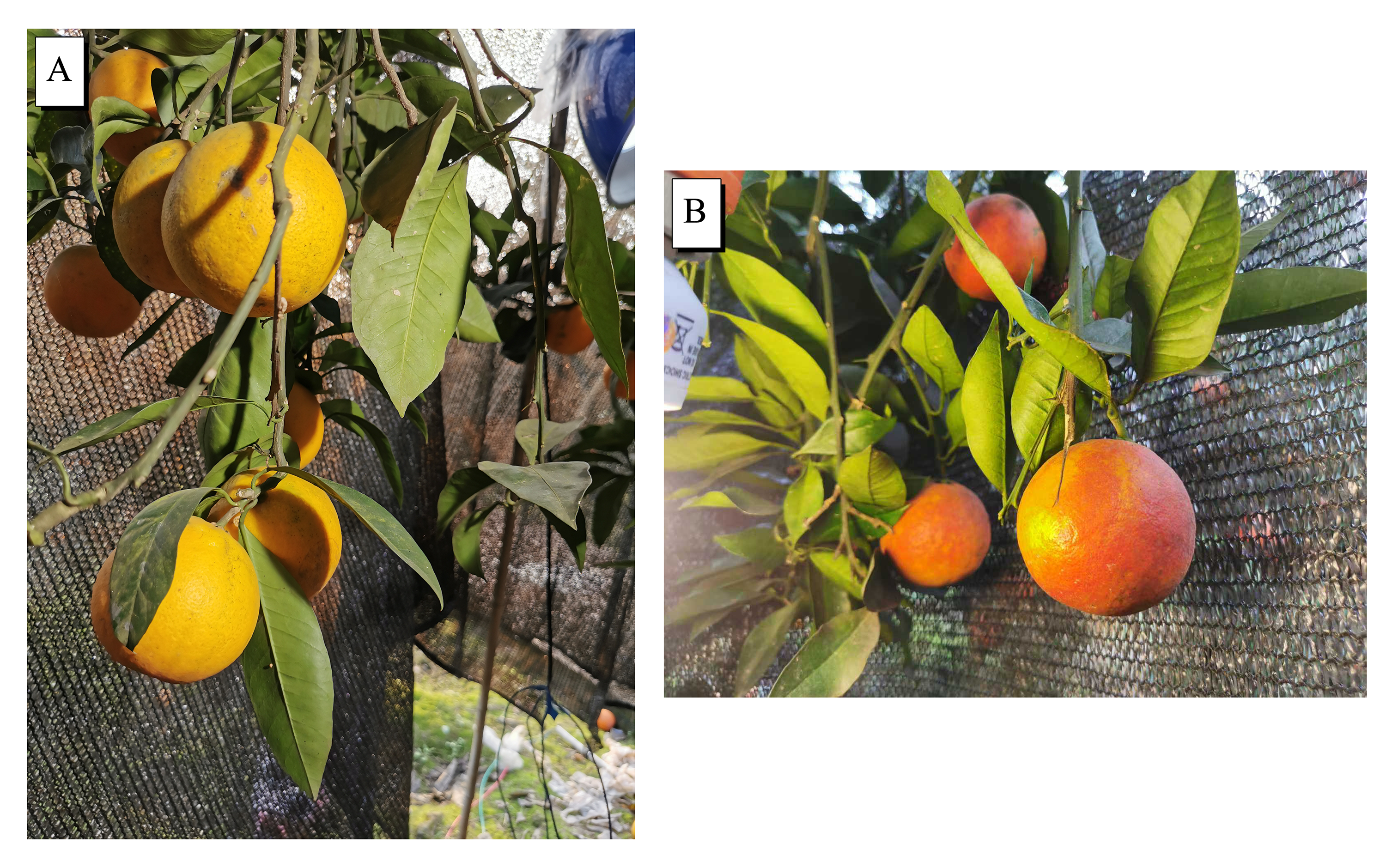


**Fig. S1 T**he on-site images of the experimental light treatments. (A) VL treatment (B) UV treatment.


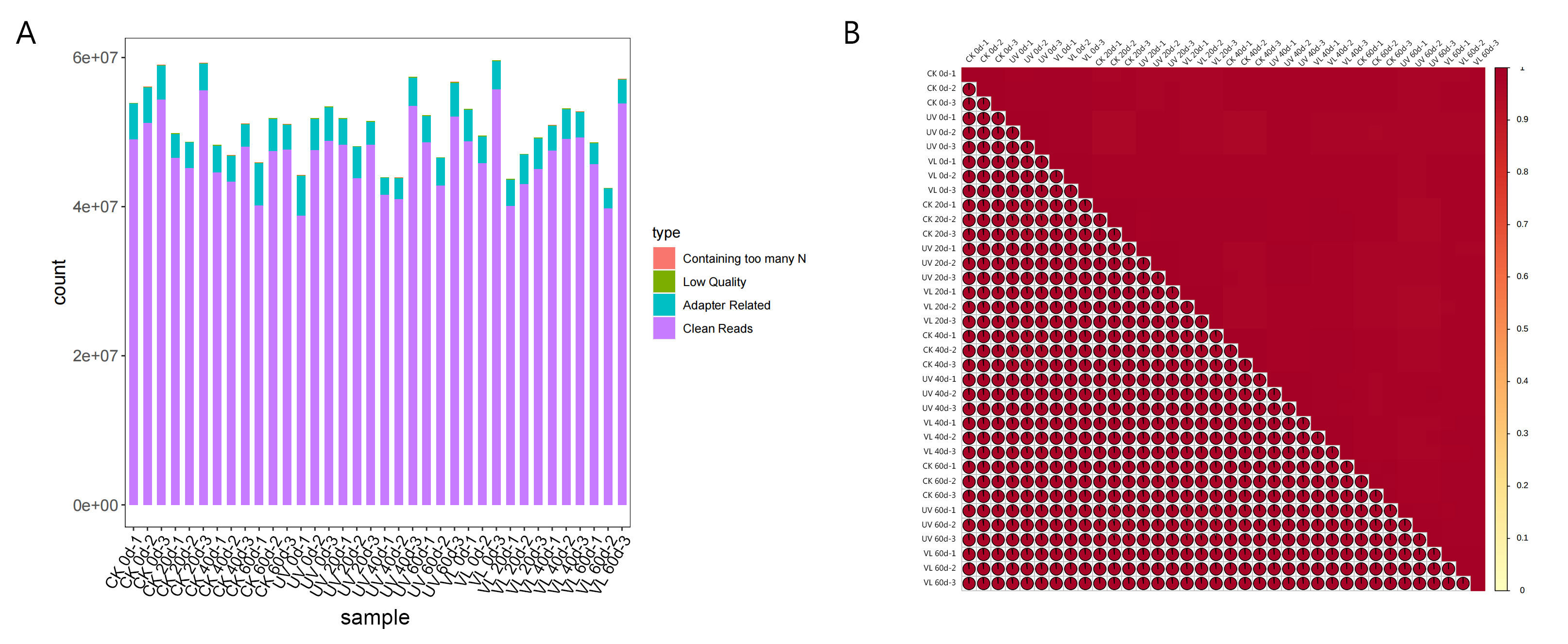


**Fig. S2** Sequencing data quality control. (A) Sequencing output statistics (B) Correlation analysis among all samples.


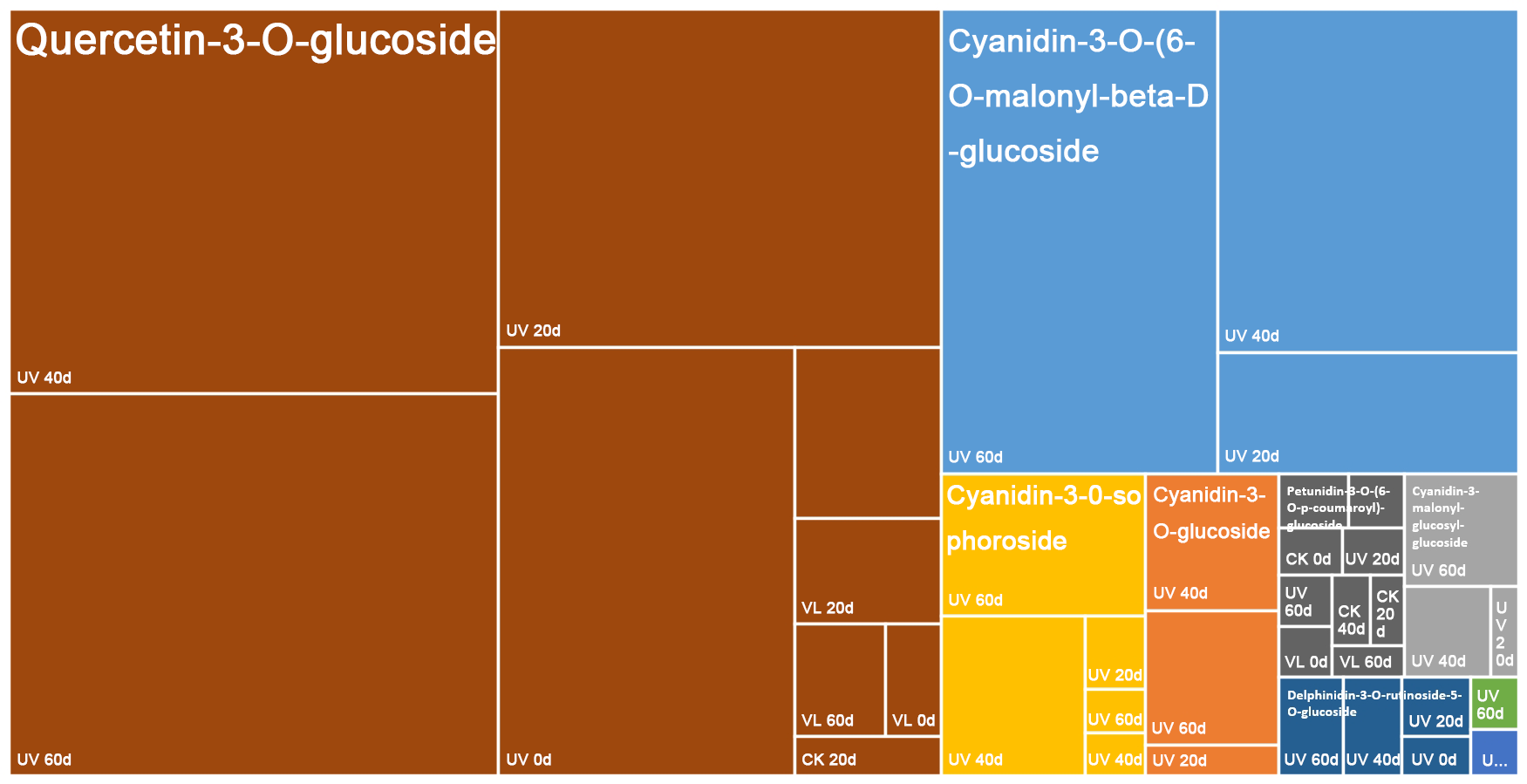


**Fig. S3** The distribution of the anthocyanin-related metabolite content in different treatment. The size of the color block represents the content of anthocyanin related metabolism


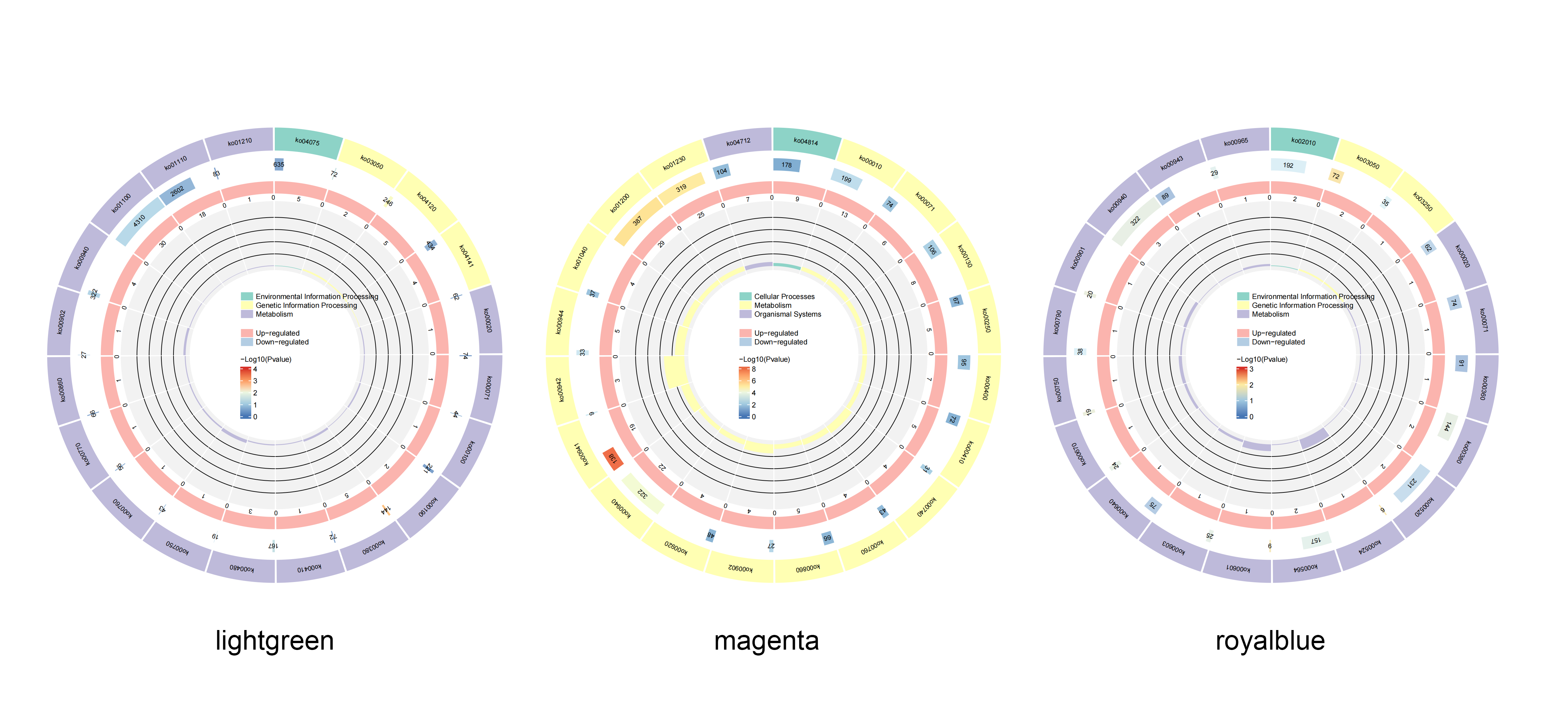


**Fig. S4** Circle charts of KEGG enrichment. The pathway of the first 20 enrichments are shown by the outer circle (first circle). Different A classes are distinguishable by their different colors. The Q values and the number of pathways in the differential background of the studied genes are shown in the second circle. The more length of the bars and their darker color intensity indicate smaller Q values and the larger number of genes in the background, respectively. The third circle is a bar chart showing the proportion of up- (Light red) and down-adjusted (pale blue) differential genes that have been adjusted up and down

**
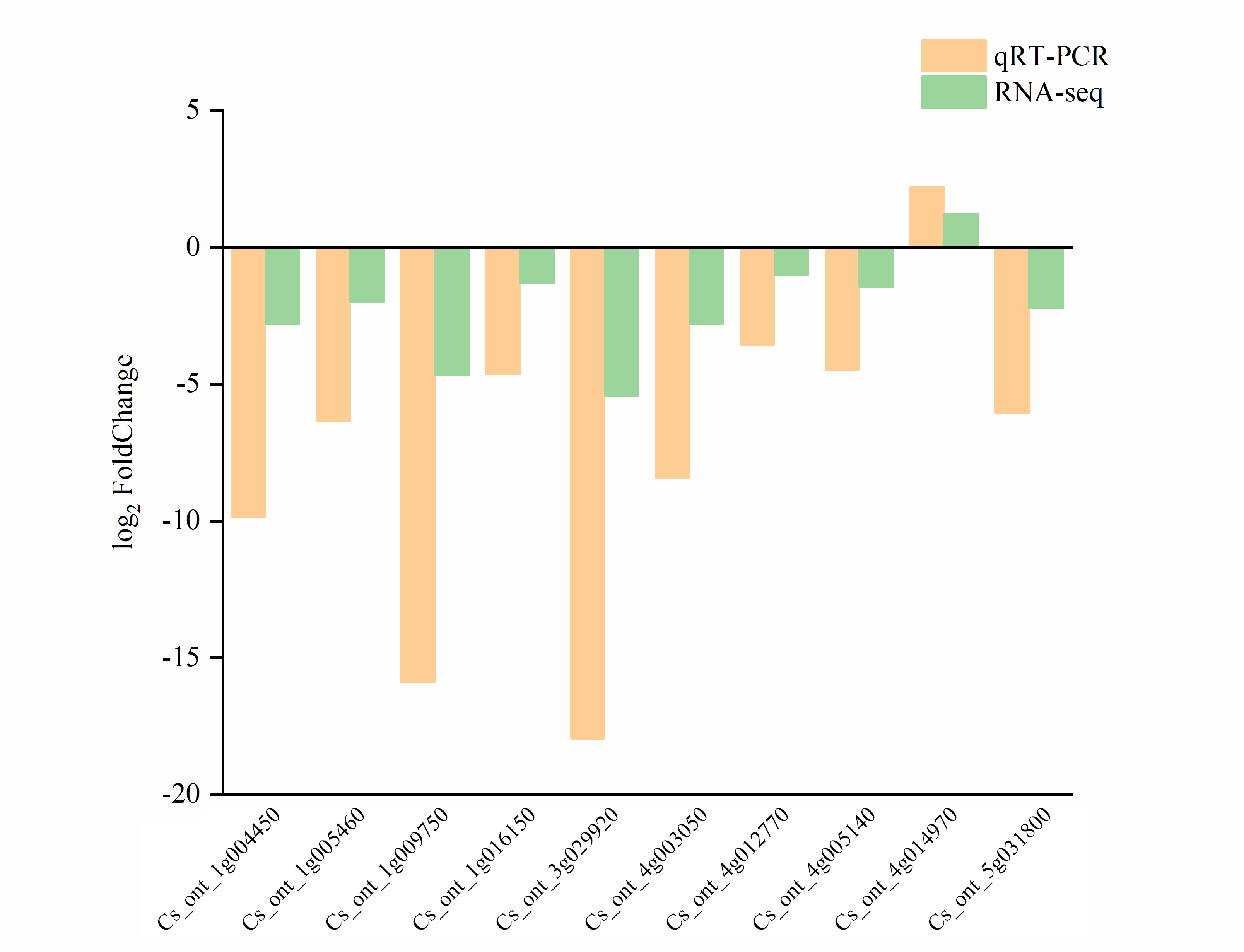

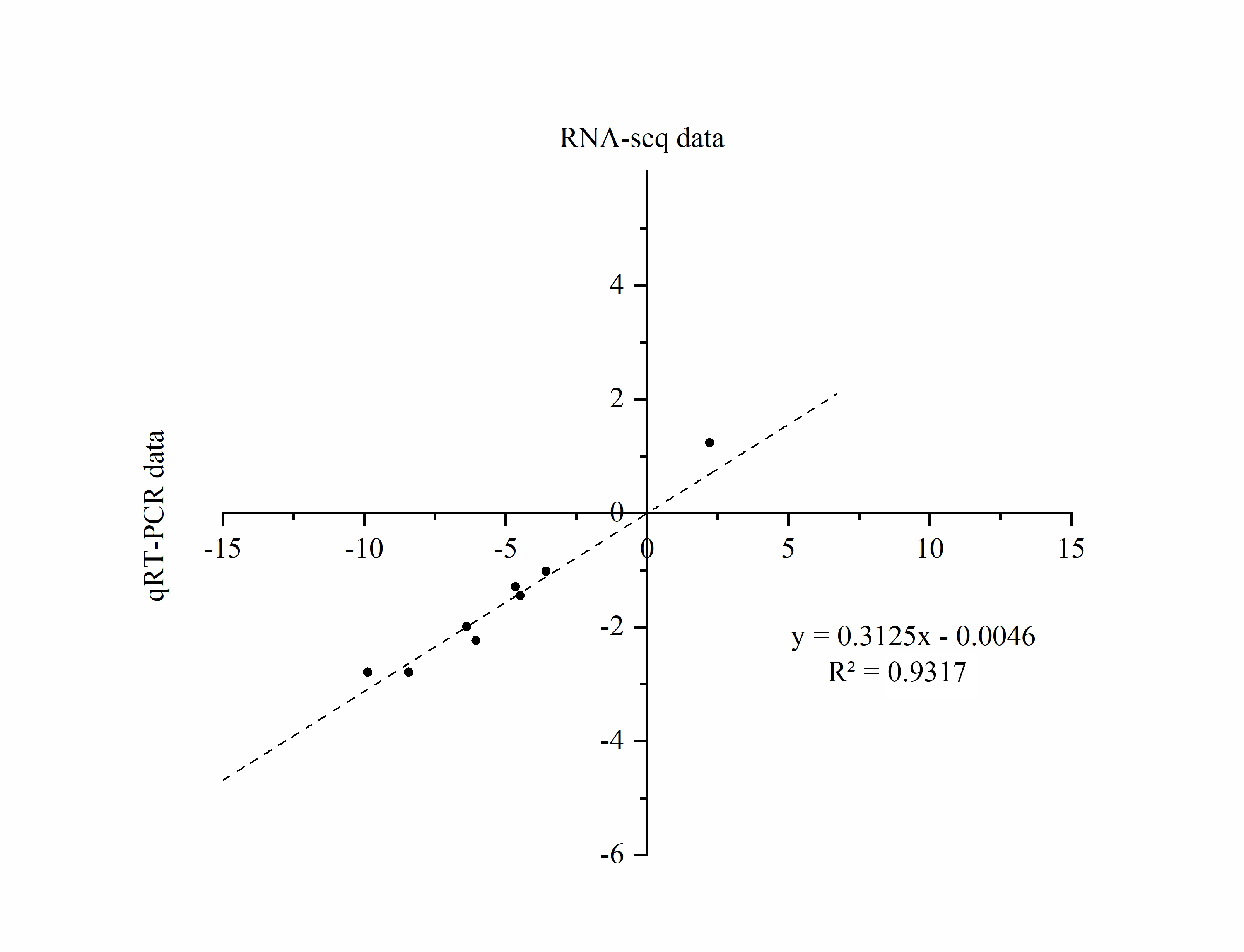
**

**Fig. S5** qRT-PCR results of the selected genes and correlation between transcriptome data and real time PCR results.


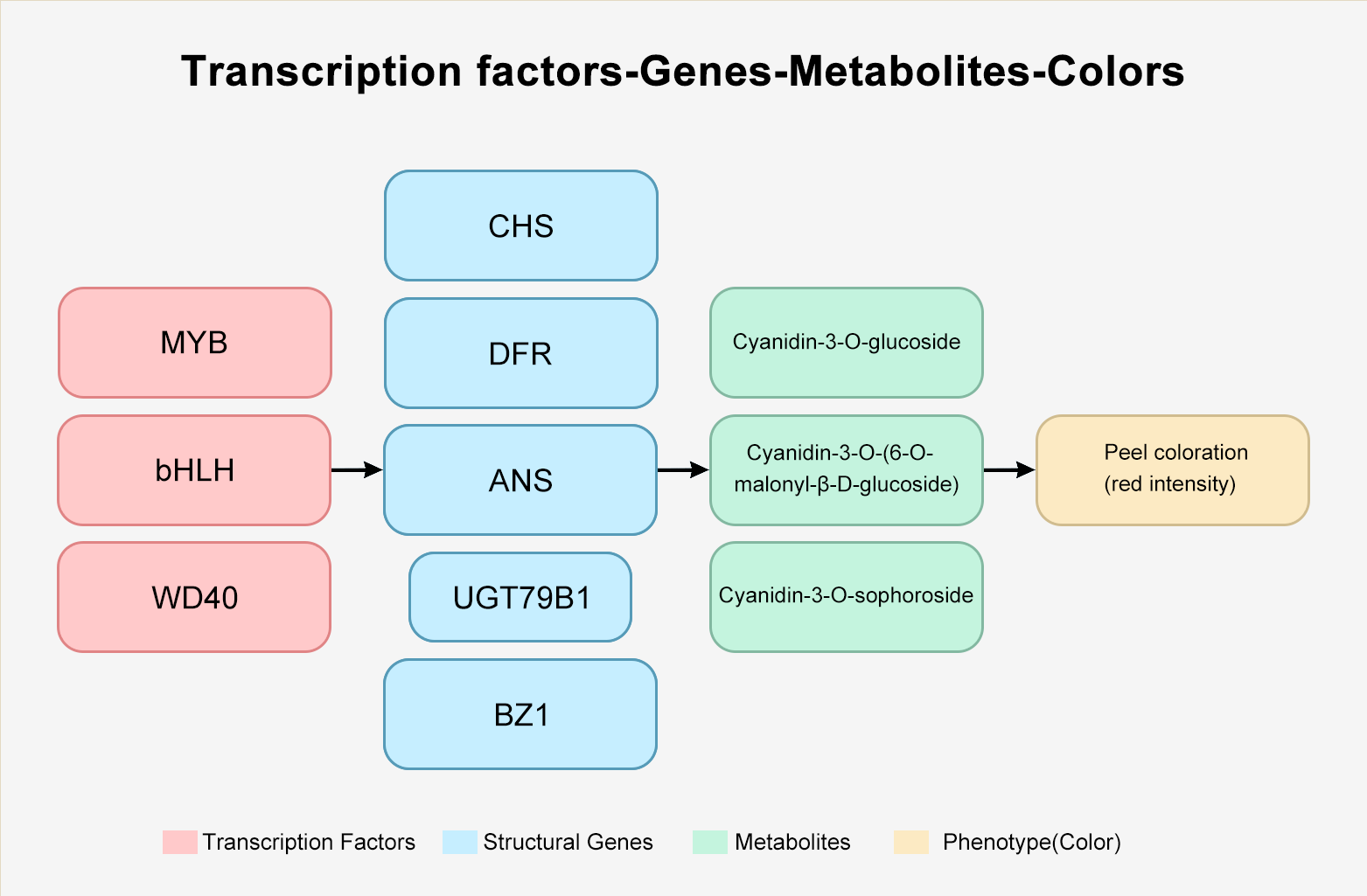


**Fig. S6** Schematic representation of the “transcription factors–genes–metabolites–colors” axis in blood orange peel under UV treatment. Arrows indicate regulatory directions along the axis.
